# Early outcome in endoscopic extended endonasal approach for removal of supradiaphragmatic craniopharyngiomas: a case series and a comprehensive review

**DOI:** 10.2478/raon-2013-0036

**Published:** 2013-07-30

**Authors:** Roman Bosnjak, Mitja Benedicic, Alenka Vittori

**Affiliations:** 1 Department of Neurosurgery, University Medical Centre, 1000 Ljubljana, Slovenia

**Keywords:** extended endoscopic approach, craniopharyngioma, trans-sphenoidal approach

## Abstract

**Background:**

The choice of endoscopic expanded endonasal approach introduces the possibility of improved gross total resection of craniopharyngioma while minimizing surgical morbidity in a significant subset of patients.

**Methods:**

From our trans-sphenoidal surgical series of 331 cases, we retrospectively reviewed visual, endocrine and neuro-cognitive outcomes in the first consecutive eight patients (median age 63 years; range 47–73 years) with newly diagnosed supradiaphragmatic craniopharyngioma (median tumour height 23 mm; range 15–34 mm), removed by expanded endonasal approach (median follow-up 27 months; range 10–69 months). Gross total resection was attempted in all patients.

**Results:**

Gross total resection was achieved in 6 of 8 patients. Visual improvement was present in 6 of 8 patients of patients or in 14 of 16 eyes. New endocrinopathy, including diabetes insipidus, appeared in 5 of 8 patients. Stalk was preserved in 4 patients. Cognitive decline was present in 2 cases. Five of 8 patients retained previous quality of life.

**Conclusions:**

Our early outcome results are comparable to the recent few expanded endonasal approach series, except for the incidence of new endocrinopathy and cerebrospinal fluid leak rate. This was influenced by higher number of transinfundibular tumours in our series, where stalk preservation is less likely, and not using nasoseptal flap or gasket closure in the first half of cases. Including data from the literature and ours, expanded endonasal approach shows a trend for improved gross total resection rate with less morbidity, more obviously for visual outcome and quality of life than for endocrine outcome. However, validity of expanded endonasal approach should be confirmed in a larger number of patients with a longer follow-up period.

## Introduction

Surgical treatment of craniopharyngiomas is a challenging issue owing to the delicate anatomical position related to the hypophyseal stalk and infundibulum and adherence to the important neurovascular structures in the suprasellar and retrochiasmatic region.^1^–^8^ The current consensus about the optimal treatment strategy has reverted in the last 2 decades back to gross total resection (GTR) in contrast to more limited surgery, aimed at tumour debulking, decompressing the optic pathways and releasing cerebrospinal fluid (CSF) pathways, followed by radiotherapy (XRT).^4^,^9^–^11^ Despite similar tumour control rates are reported with subtotal resection and adjuvant XRT, the surgery for recurrence is associated with unacceptably increased morbidity and mortality rates.^10^–^12^ The challenge of more favourable long term recurrence rates in GTR is faced by increased risk of visual, pituitary and hypothalamic dysfunction; only pituitary insufficiency is nowadays acceptable with modern hormonal substitution therapy. Several transcranial approaches have been established for maximum resection of craniopharyngiomas, many of them extending far beyond the sella.^9^,^13^–^17^ In these principally lateral approaches, the direct visualization of suprasellar-retrochiasmatic region is incomplete on the contralateral side, resulting in a significant amount of blind dissection. The first 100 patients, published to date in a few series, have been operated on using endoscopic transplanum-transtuberculum approach for newly diagnosed or residual and recurrent supradiaphragmatic craniopharyngiomas.^18^–^24^ The choice of endoscopic extended endonasal approach (EEA) as the minimally invasive access introduces the possibility of complete tumour resection while minimizing surgical morbidity in a significant subset of patients.^2^,^18^–^20^,^22^,^23^ This midline approach has the advantage of providing direct visualisation of the suprasellar and third ventricle tumour extension and visually controlled in-line dissection at the plane between the tumour and the optic nerves, chiasm, stalk (if recognizable), hypothalamus and supraclinoidal internal carotid arteries with their branches.

Improved GTR and less morbidity with EEA compared to transcranial surgery has not yet been proved as the number of publications on EEA is limited. The aim of this report is to contribute our experience in the endonasal endoscopic treatment of newly diagnosed supradiaphragmatic craniopharyngiomas with special attention to stalk preservation and postoperative visual, endocrine and cognitive/social outcomes. Results are compared to the recent series and the utilities and limitations of the EEA approach in supradiaphragmatic craniopharyngiomas are extracted.

## Patients and methods

From our trans-sphenoidal series of 331 cases, we retrospectively reviewed records of 112 cases of patients with suprasellar lesions, treated with EEA at the Department of Neurosurgery, University Medical Centre Ljubljana, Slovenia (January 2007 – December 2011). The analysis revealed 8 consecutive adult patients with pure suprasellar craniopharyngioma that were treated with endoscopic EEA. GTR was attempted in all patients. All patients gave their informed consent and all surgeries were performed by the senior author (R. B.).

Patients underwent a comprehensive ophthalmological evaluation at the University Eye Clinic pre- and postoperatively, consisting of the assessment of visual acuity, visual field (Goldmann) and fundoscopy.

Endocrine evaluation was performed at the Department of Endocrinology at the University Medical Centre Ljubljana. Patients were evaluated with basal levels of prolactin (PRL), thyroid-stimulating hormone (TSH), free triiodothyronine (FT3),, free thyroxine (FT4), growth hormone (GH), insulin-like growth factor 1 (IGF-1), luteinizing hormone (LH), follicle-stimulating hormone (FSH), cortisol, testosteron (free, total) and adrenocorticotropic hormone (ACTH) test. If necessary, diabetes insipidus (DI) was diagnosed according to the freezing point depression osmometry of urine, fluid intake, urine volume and osmolality of urine (Table 1).

One patient (Patient 3) presented with moderate psychoorganic change, headache and balance problems from slowly developing hydrocephalus in the last 4 months. One patient (Patient 4) was wrongly diagnosed with a large pituitary adenoma 2 years ago in a peripheral hospital and denied surgery. She received complete hormonal substitutional therapy, but was referred to our department because of progressive visual loss and psychoorganic change during the last 4 months.

**FIGURE 1.**
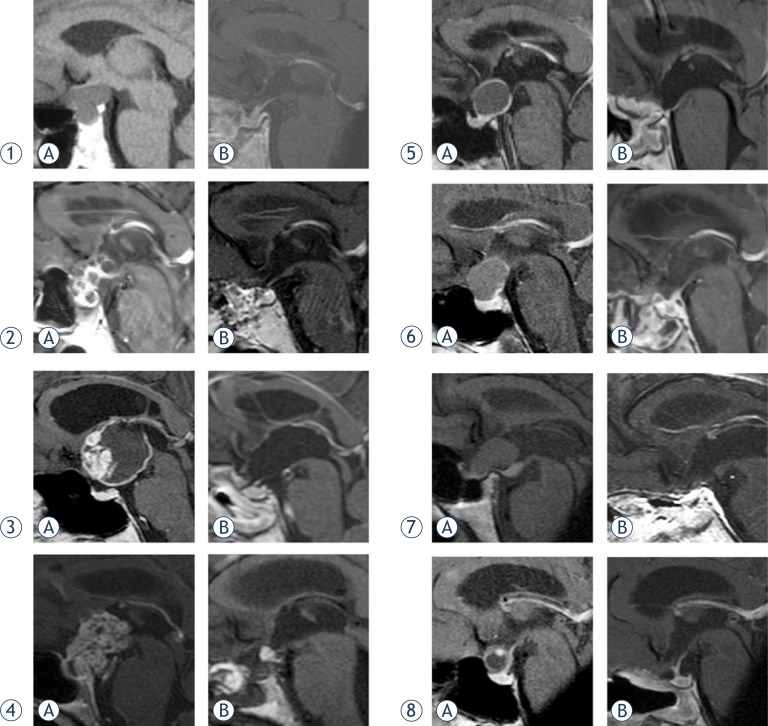
MRI scans (sagittal planes) of craniopharyngiomas in eight patients preoperatively **A** and postoperatively **B**.

Postoperatively, hormonal status was evaluated at 3, 6 and 12 months and then yearly. Further endocrinological evaluation sheme was modified according to the presence of any hypothalamic-pituitary dysfuction. The extent of resection was independently evaluated by neuroradiologists comparing pre- and postoperative magnetic resonance images (MRI). T1-weighted contrast-enchanced, T2-weighted and three-dimensional (3D) magnetization-prepared rapid gradient-echo images (MP-RAGE) were used for pre- and postoperative evaluation. Individual preoperative and postoperative MRI scans (sagittal planes) of craniopharyngiomas in 8 patients are presented in Figure 1.

### Surgical technique

Under general anesthesia, the patient is placed in the supine position on the operating table with the head fixed into anteflexion for 30° and rotated toward the right shoulder for 30°. In patients with a short neck, the shoulders and the upper back are supported with a cushion to achieve the desired neck anteflexion without jugular vein compression.

Patient’s head is first registered with the neuro-navigation device (Stealth Station 5.0, Medtronic, MN, USA).

The nostrils are washed with anstiseptic solution and temporarily packed with cottonoid strips soaked with 10% Xylocaine. The neurosurgeon is positioned face-to-face with the patient’s right side while the assistant is at the right lateral side of the patient’s head. The endoscopic tower with the high-resolution screen is positioned 1 m left lateral to the patients head in the line of sight of the neurosurgeon and the assistant. The scrub nurse is positioned on the left side of the patient. The binostril approach is more comfortable for the surgeon and provides less instruments collision with the use of 2 nostrils-four hands technique. A 0° and less frequently 45° or 70° rigid endoscope (180/4 mm) is used (Karl Storz, Tuttlingen, Germany).

### Nasal phase

The endoscope is introduced into the left nasal cavity first. The middle turbinate (MT) is gently lateralized and the space between the MT and the nasal septum is gently widened. Only large and pneumatized MT is removed. The base of the MT, vomer and nasal septum are infiltrated with adrenaline (diluted 1:50.000) to promote vasoconstriction. The choana is identified and then sphenoidal opening is identified in superior direction about 1 cm above the inferior margin of MT and medial to the superior turbinate. A vascular pedicle nasoseptal flap is formed, turned inferiorly into the choana and saved for the subsequent sellar floor reconstruction.

On the right side, however, MT is only lateralized. The posterior third of septal mucosa is removed. A large opening is made in the posterior third of the denuded nasal septum. The lamina perpendicularis is removed in one large piece if possible and stored for sellar reconstruction. The endoscope is inserted through the right nostil and held by the assistant, while the neurosurgeon works through both nostrils bimanually, holding aspirator in the left hand and the drill handle, or bayonet microdissector or other fine instruments in the right hand.

### Sphenoidal phase

The anterior wall of the sphenoidal sinus is widely opened and the septa removed. Inside the sphenoidal sinus, the sella is then localized. The posterior sphenoidal wall is denuded of the mucosa, which is removed or rolled in the lateral and the bottom part of the sphenoid sinus. Visualization of the sellar region is initially performed with a 0° endoscope. Sellar floor, bilateral protuberances of the anterior loop of the internal carotid arteries, major and minor optocarotid recesses (OCR), optic canals and paraclival carotid columns are identified. The use of neuronavigation in sphenoidal sinuses with atypical or blurred morphology, or occasionally with thick posterior wall is beneficial.

The remnants of the posterior ethmoidal cells, crushed lateraly from mucosal side, are gently punched off for sufficient midline exposure of the sphenoid ceiling and OCRs.

### Sellar and planar phase

The sellar floor is opened with a highspeed drill or Kerrison rongeur. The blue line of the superior intercavernous sinus marks the transition from the anterior sellar wall to the planum sphenoidale. Here, the bone is removed in the superior direction for 1.5–2 cm and superolateral to OCR. The drilling around OCR and proximal parts of optic canals is gentle, intermittent and performed with constant irrigation of the diamond drill bit with cold saline to prevent the optic nerve from thermal injury.

The sellar and planar trephination together constitute the so-called “chief’s hat” exposure of dura. This is generally sufficient to expose the planar dura up to the line of the dura propria traversing between the optic nerves.

The dura, exposed by the “chief’s hat” trephination, is incized in T-shape: horizontally along the dura propria or a little above it, and vertically across the superior intercavernous sinus and along the midline over the pituitary and continued horizontally along the sellar floor on each side. The dural flaps are turned laterally (Supplement 1).

### Intradural phase

When the arachnoid is dissected, CSF starts to leak and the dome of the craniopharyngioma is exposed, pushing up the chiasm and the optic nerves and displacing downward the remnants of the diaphragm sellae (in pure suprasellar craniopharyngiomas) and the pituitary. The cyst of the craniopharyngioma is punctured and the dark oily liquid pours out, immediately decompressing the chiasm and the optic nerves (Supplement 2).

**FIGURE 2.**
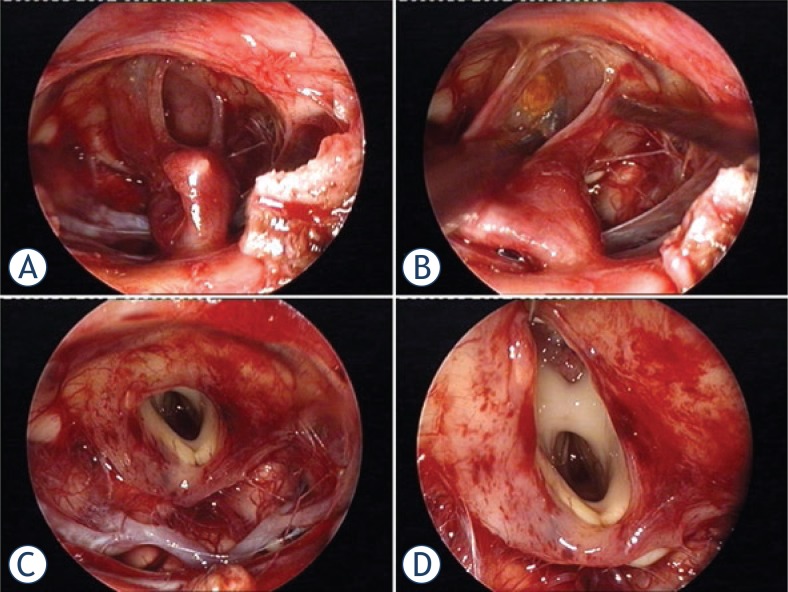
In this cystic craniopharyngioma (Patient 5), the stalk was centrally infiltrated close to the pituitary and could not be preserved **A**. The incipient third ventricle entrance is seen from intracavitary view. The slit into the third ventricle is still covered with tumour capsule **B**. Complete removal of the capsule opened the third ventricle **C**. Petehiae in the hypothalamus bilaterally resulted from apparently gentle traction and blunt dissection of the capsule away from the hypothalamus **D**. Psychoorganic change, disorientation and memory deficits were noticed in less than a week after surgery, the transient sleep disorder become apparent in the second week postoperatively (see also a supplemented video material 2).

Sometimes, the cyst is filled with cholesterine cristals. If the tumour is solid and calcified, it is first debulked. Tumour is entered by the longitudinal incision. The large calcifications have sometimes to be crunched first by micro-roungeur into smaller pieces. The solid part of the tumour with calcinations is removed by piecemeal technique, bearing in mind the stalk position when recognizable. The tumour is dissected extracapsularly in the clockwise direction along the tumour circumference from the superficial to the deep seated suprasellar structures. Following the arachnoid-tumour plane, the tumour capsule is detached first from the right supraclinoidal internal carotid artery (ICA) and the right optic nerve, from the undersurface of the chiasm, then from the left optic nerve and left ICA. Special attention is paid to perforators of the chiasm, hypophyseal and Dawson’s arteries, originating from the supraclinoidal ICA. The striate structure of the stalk is carefully searched for around the surface of the cyst or grossly debulked tumour and from the inside aspect of the cystic craniopharyngioma. In supradiaphragmatic craniopharyngiomas, the stalk is first identifiable inferiorly at the pituitary, but should be traced up to the hypothalalmus and at least partially preserved if possible (Figure 2). This condition is rarely present (Supplement 3).

**FIGURE 3.**
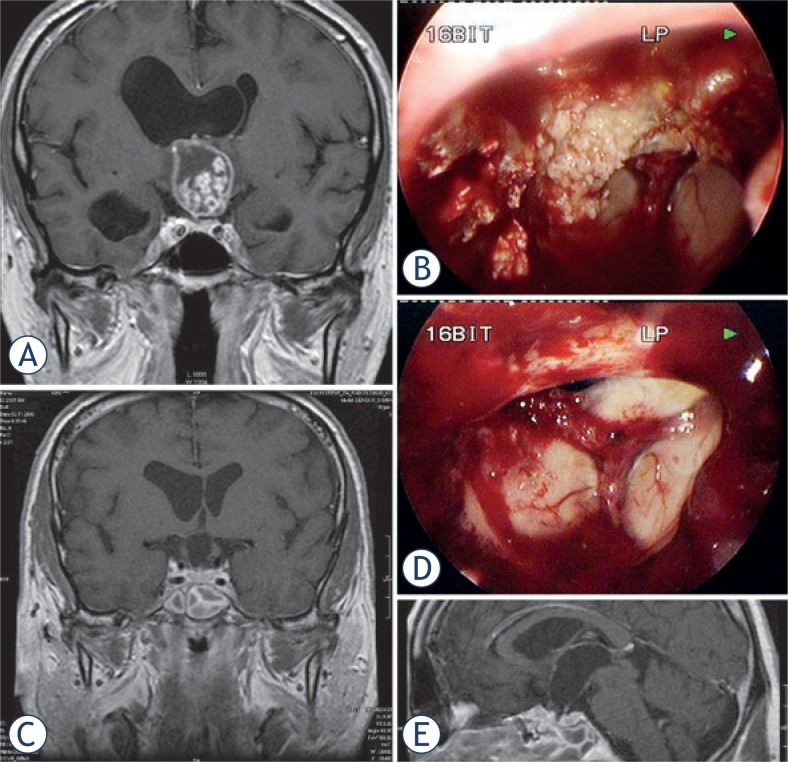
Large craniopharyngioma (Patient 3) produced unilateral hydrocephalus by obstructing the right formen of Monro **A**. The dome was filled with soft cholesterine cristals **B**, which were easily removed. Lower limbus of the right foramen of Monro is seen through the empty third ventricle **D**. Despite bilateral preservation of anteromedial hypothalamus **C** and stalk preservation **E**, the patient developed panhypopituitarism and diabetes insipidus with long lasting psychoorganic change.

The surgeon must soon get the idea of a tumour growth type with stalk expansion (transinfundibular) or stalk dislocation (pre- and retroinfundibular). The stalk points toward the hypothalamus and the third ventricle floor, which may be displaced and distorted in extraventricular tumours or entered and split in extra- and intraventricular tumours.^2^,^6^ The continuity of the stalk with the hypothalamus will result in functional integrity. In middle-sized and large transinfundibular tumours, the hypothalamus is split and tumour protrudes into the anterior third ventricle up to the foramina of Monro (Figure 3). Here the tumour is most adherents to the inner walls of infundibulum, which are most often dislocated inferolaterally. Meticulous sharp dissection of the tumour is necessary here at the exact tumour-glial plane.^3^ Infiltration or severe adherence to the hypothalamus in the deep aspect of the dissection preclude partial resection to prevent hypothalamic injury (Figure 2). The rest of the tumour inside the third ventricle will be easier to detach from ventricular wall when infundibulum is passed. When the tumour is removed from the retrosellar region, corpora mamillaria, tip of the basilar artery, posterior cerebral arteries – P1 segments and the oculomotor nerves and the Liliequist’s membrane will be exposed (Supplement 2).

Following tumour resection, both 0° and angled endoscopes are introduced into the surgical cavity to explore for any residual tumour (Figure 3).

**FIGURE 4.**
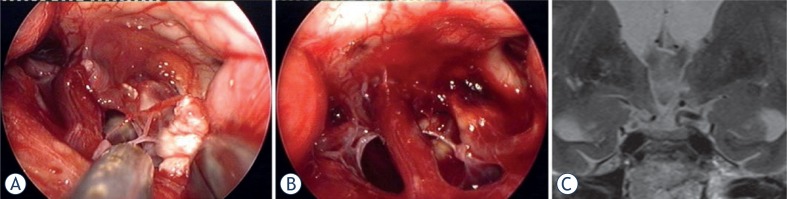
The capsule of the cystic craniopharyngioma was firmly attached to the left hypothalamus, the stalk was dislocated to the right side (Patient 6). The outgrowth of the craniopharyngioma from proximal stalk is recognizable **A**. Complete removal of the capsule was possible, but produced subpial blood injection over the left hypothalamic surface **B**. MRI scan revealed a small ischemic injury in the left hypothalamus **C**. This patient had transient sleep disorder, moderate hyperphagia and memory problems (see also a supplemented video material 1).

In more favourable cases, the craniopharyngioma grows exophytically from the stalk, which can be partially preserved with careful dissection and avoiding traction. In true extraventricular craniopharyngioma, tumour can be severely adeherent to the the outer pial surface of the hypothalamus. Traction to the hypothalamic parenchyma and hypothalamic perforators should be avoided as much as possible (Figure 4, Supplement 2, Supplement 3).

For the reconstruction of the sellar floor, we use facia lata (FL) and Hadad’s flap in a three-layer technique (suprasellar fat packing + inlay of FL + onlay FL + Hadad’s flap + sealant) and gelfoam or fat are used as a buttress by packing the posterior sphenoid sinus (Supplement 1).^25^,^26^ Lumbar drainage is applied postoperatively for 7–10 days.

## Results

Eight patients were enrolled in the study (male:female = 4:4; median age 63 years; range 47–73 years). The follow-up period was 10–69 months (up to 5 years and 9 months, median 72 months).

Seven of 8 patients presented with a visual problem as a leading symptom, median duration of visual symptoms was 5 months (range 6–2 months). Except in Patient 4, there was no preoperative endocrine dysfunction in the other 7 patients and none of the patients had DI. The median tumour height was 23 mm (range 15–34 mm), six were equal or taller than 20 mm. All tumours were of adamantinomatous type.

Visual, endocrine, neurological and cognitive outcomes in 8 patients with craniopharingioma are revealed in Table 2.

Visual field defects were bilateral in 6/7 patients with visual disturbance (Table 1). Visual improvement was estimated according to the postoperative ophthalmological evaluation and subjective information from the patient. Visual status has improved, normalised or remained normal in 6 of 8 patients postoperatively or in 14/16 eyes; in 2 patients, improvement was unilateral only and new visual field deficits appeared in the other eye. In one patient (Patient 7), new visual field deficit appeared in a delayed fashion after initial improvement (on the postoperative day 4). There was no decline of visual acuity and all patients recover the reading capacity with correction (Table 2).

Stalk preservation was possible in 4/8 patients (Table 2). In 2 of them, endocrinological evaluation revealed normal pituitary function, in one panhypopituitarism (1/3 of the stalk thickness preserved; Patient 7) and in one partial hypopituitarism (substitution of tyroid hormon and ADH necessary, substitution of cortisol abandoned after 6 months postoperatively; Patient 6). The rates of DI and new endocrinopathy were 5/8 patients (Table 2).

Hypothalamic dysfunction was initially noted in 4 patients (Patients 3–6), but remained persistent in 2 patients (Patients 3 and 6). Short term memory deficits, confusion and spatial disorientation were recognized early within the first postoperative week. Three patients suffered from hyperphagia and gained 15–30 kg in the first 12 months postoperatively before stabilizing the body weight. Sleep rhythm was temporarely disturbed in 2 patients. It was observed in the second postoperative week and subsided spontaneously after 2–3 months. The psychoorganic syndrome was mild and transient in 2 patients (Patients 4 and 5) and more significant in another 2 (Patients 3 and 6). Memory deficit and emotional lability started to improve after one year as reported by the relatives, but were still observable in the follow-up period (Table 2).

The quality of life was retained in 5/8 patients. It was decreased in 2 patients due to cognitive problems (Patients 3 and 6) and in one due to new visual field deficit on one eye (Patient 7).

Despite the attempt at GTR in all patients, near total (>95%) removal was achieved in one patient (in Patient 7, a translucent capsule of 1.5×1 cm^2^, firmly adherent to hypothalamus, was left in place; Figure 1 (1–7b)) and subtotal removal (approx. 90%) in one patient (0.6 cm midline residual tumour in the optic recess was left in Patient 4, Figure 1 (1– 4b)). GTR rate was achieved in 6/8 patients. None of the patients was referred for adjuvant XRT during the present follow-up period. In Patient 7, MRI after 7 months shows no tumour and no capsule, but some microcalcinations. In Patient 4, the sub-centimeter residual tumour shows no progress (Figure 1).

The evolution of the sellar closure technique, implanted materials and exact closure in individual patients are revealed in Table 3.

The lumbar drainage was preventively inserted in all patient for the arbitrary 10 days. CSF leak reappeared in 2 patients after 11 (Patient 4) and 20 (Patient 5) days. The revision included an attempt to identify the leakage point using fluorescein dye and tamponade it with fat, followed by sphenoidal sinus obliteration. However, in Patient 4 a second revision was necessary with completely new sellar closure with bilayered fascia lata and a sealant (Table 3). Both patients suffered meningitis from prolonged CSF leak and lumbar drainage and were treated with antibiotics and needeed ventricularperitoneal (VP) shunting.

In the first patient with CSF leak (Patient 4), the synthetic inlay of Neuropatch (B. Braun Melsungen AG, Melsungen, Germany) was combined with Tachosil (Takeda Pharmaceuticals International GmbH, Zurich, Switzerland) and obliteration of sphenoid sinus with Spongostan™ Absorbable Haemostatic Gelatin Sponge (Ethicon Biosurgery, Somerville, New Jersey, USA) and glue (Beriplast, CSL Behring, King of Prussia, Pennsylvania, USA).

In the second patient with CSF leak (Patient 5), the synthetic inlay (Neuropatch) was combined with Hadad’s flap which did not completely cover it, resulting in a leakage point. Althrough efficient closure technique was reported in 2006, it was not usen in the first patients (Table 3).

The bad experience with these 2 patients precluded the use of autologous material (fascia lata) in bilayered sellar closure technique in combination with Hadad flap and sealant Duraseal™Exact (Covidien, Dublin, Ireland) in the following patients. In the last 3 consecutive patients there was no CSF leakage using Hadad’s flap. In the last patient, only synthetic and heterologous material was successfully used in combination with the Hadad’s flap (Table 3).

## Discussion

Craniopharyngiomas are slowly growing epitelial tumours, originating from the epithelial remnants of the craniopharyngeal duct or Rathke’s pouch (adamantinomatous subtype) or result from the metaplasia of squamous epitelial cell remnants (squamous papillary form).^12^

Craniopharyngiomas have bimodal incidence with the first peak in children at 5–14 years and second in adults at 65–75 years. Clinical signs and symptoms are diverse and usually appear insidiously. Bitemporal hemianopsia from inferior compression of the chiasm is a typical visual disturbance. Endocrine dysfunction varies from indirect hyperprolactinemia, partial hypopituitarysm and panhypopituitarysm to precocious puberty in children. Large tumours can obstruct CSF pathways and produce obstructive hydrocephalus or affect the hypothalamus. Obesity, sleep disorders, apathy, emotional lability, short-term memory and thermoregulatory problems may accompany the altered neuropsychological profile and decrased mental performance at job or at school. Hydrocephalus and visual deterioration may also have an acute onset. The spontaneous rupture of cysts, containing dark oily liquid, may produce chemical meningitis.^4^,^12^

The radiological appearance of craniopharyngioma is most commonly that of a cystic/solid and calcified lesion in the suprasellar region. They physically originate from the stalk or the infundibulum. In contrary to pediatric population, only a small number of tumours in adults have infradiaphragmatic origin.^27^ The extrasellar growth patterns are numerous.^6^,^9^,^13^–^15^,^17^,^28^

As the hypothalamic involvement is the single most important predictor of completness of removal and precludes surgical morbidity, MRI is the imaging method of choice to display dislocation or invasion of the hypothalamus in suprasellar retrochiasmatic growth.^3^–^6^,^8^ However, the exact outgrowth of craniopharyngioma from the stalk or the infundibulum can not be precisely anticipated from MRI scans.^4^–^6^

### Grading of craniopharyngiomas

Several grading systems were introduced to aid neurosurgeons in planning their surgical strategy either preoperatively from MRI scans and/or judging the feasibility of preservation of stalk and hypothalamus intraoperatively.^2^,^3^,^6^–^8^,^13^,^17^,^29^ Hoffmanm proposed pre-, sub-, retrochiasmatic and intraventricular craniopharyngiomas.^13^ Intra- or suprasellar, extra- or intraventricular tumour have been proposed by Yasargil, Wang and Steno.^6^,^7^,^8^,^17^ Samii *et* Tatagiba proposed the following grading scale: I – intrasellar or infra-diaphragmatic, II – occupying the cistern with or without an intrasellar component, III – lower half of the third ventricle, IV – upper half of the third ventricle, and V – reaching the septum pellucidum or lateral ventricles.^16^ Kassam based his classification on the infundibulum: preinfundibular, trans-infundibular, or retroinfundibular and isolated intraventricular.^2^ Qi *et al*. studied the arachnoid envelope around the stalk.^5^ They noticed that the arachnoid is more firmly attached to the proximal than distal stalk and divided craniopharyngiomas according to the 4 basic growth patterns: infradiaphragmatic, extraarachnoidal, intraarachnoidal and subarachnoidal. According to MRI analysis of 195 cases, these theoretical growth patterns were combined into 5 distinct locations of craniopharyngioma expansion: infradiaphragmatic, extraventricular, extra- and intraventricular, transinfundibular and infundibulo-tuberal.^5^

The protective role of the diaphragm sellae in craniopharyngiomas with infradiaphragmatic origin and suprasellar-retrochiasmatic extension is responsible for less hypothalamic morbidity because such tumour growth is essentially extra-arachnoidal.^7^,^8^,^27^ In supradiaphragmatic craniopharyingiomas, the classification to extra- and intra ventricular and mixed-type has important clinical implications.^3^,^6^,^7^ Steno *et al*. found in all 25 mixed tumours and in 3/18 considerably extraventricular tumours a strip of firm adherence to the third ventricle walls around the equator of the tumour, separating extra- and intraventricular portions.^6^ The tumour-glial interface, recognized by many authors and confirmed histologically, may provide a thin nonfunctional cleavage line which may preserve the function of the nuclei and alleviate hypothalamic morbidity.^5^,^30^ The EEA may provide superior results in transinfundibular craniopharyngioma by enabling bilateral dissection at the cleavage line around the tumour circumference under direct vision and in the line of growth, with no blind retraction of the capsule from the hypothalamus on the contralateral side, as present in the lateral transcranial approaches, or upward, as present in transcallosaltransforminal removal. However, the preservation of the morphological integrity of the hypothalamic floor and the stalk is very rarely possible and does not guarantee the function (Figure 3).

We tried to grade our cases according to different classifications and compared the grade with our intraoperative findings (Figure 1, Table 1). None of our cases had an infradiaphragmatic component, although the inferior part of the tumour protruded into the sella in 5 cases (Patients 1, 2, 4, 6, 7). There was a significant discrepancy in medium sized tumours according to false and true intraventricular extension. Solely judged by MRI, the tumour of Patient 3 might also represent pure intraventricular craniopharyngioma or infundibulo-tuberal growth.^2^,^5^ Such tumours are generally approached by transcallosal transforaminal or translaminar approach. Regardless of different classifications, MRI is often not conclusive about the stalk and hypothalamic dislocation or infiltration. The final surgical decision relies upon intraoperative details which are augmented by direct and bilateral visualization in EEA.

### Gross total removal rate

GTR is related to a favourable long recurrence-free period and even cure. About 10–15% of totally resected craniopharyngiomas recur and up to 80% are symptoms free after 10 year after GTR without adjuvant XRT.^9^,^31^–^33^

GTR of craniopharyngioma is related to the origin of growth and pial and arachnoidal planes.^5^,^30^ Virtually all craniopharyngiomas are related to the hypophyseal infundibulum and the removal of the adherent tumour is related to the high risk of hypothalamic injury (Supplements 1–3). In suprasellar extraventricular craniopharyngiomas, the surface of the tumour can be detached from the arachnoid with the preservation of subchiasmatic and hypothalamic arteries. However, when protruding inside the hypothalamus into the third ventricle, it becomes firmly adherent to its parenchyma inside the infundibulum (Figure 2b). GTR might become impossible in such cases and sharp excision should be attempted to achieve near or subtotal removal (Patients 4 and 7).

The GTR rate in our series was 75% 6/8 patients, which is a solid result compared to recent series using EEA (Table 4).^18^–^24^,^31^ We decided against immediate postoperative irradiation in Patient 4 (0.6 cm midline residual in the optic recess, old age) and in Patient 7 (only translucent capsule of 1.5 × 1 cm^2^ was left attached to the hypothalamus). None of these 2 patients with near total removal (NTR) and subtotal resections (STR) subsequently deteriorated or showed progression on MRI during the follow-up period (Table 2).

### Visual outcome

Vision improvement or normalisation is expected in most surgical cases, the rate of visual deterioration is around 15%.^31^,^33^,^34^ Vision deterioration is less likely with trans-sphenoidal surgery, especially with preoperatively normal vision.^1^,^33^

EEA offers midline panoramic direct visualisation all around the tumour and is the most beneficial in dissecting the tumour dome from the undersurface of the chiasm and optic nerves. Special attention must be paid to the perforators of the chiasm which stem from distal supraclinoid internal carotid artery (ICA) and are pushed posteriorly by the tumour (Supplements 1–3). Mechanical trauma and vascular injury from blind dissection to contralateral structures which is more likely with lateral transcranial approaches. are minimized with EEA.

All patients recovered reading capability with correction on each eye, however in 2 patients new visual field deficit appeared or enlarged in one eye (but improved in the other eye). The visual improvement rate in our series was in 6/8 (75%) patients or 87.5% of eyes, which is in accordance with the recent series (average 79.4%, range 71–93; Table 4).^18^–^24^ Vasospasm may be related to the delayed appearance of visual disturbance. After initial improvement, Patient 7 reported unilateral increase of visual field deficit on the postoperative day 4. Interestingly, central vision improved in all eyes, what may be related to the compensation by the fusion reflex of nasal halves of the visual fields.

### Postoperative endocrinopathy

Stalk sacrifice is generally an acceptable price for GTR and long lasting recurrence-free time with modern hormonal replacement therapy, and is performed by many surgeons even in cases where stalk preservation is feasible.^10^,^13^–^17^,^31^–^33^,^35^–^38^ Stalk preservation is related to 40–50% of thyroid and adrenal function.^10^,^18^,^38^,^39^

The retroinfundibular (Patient 1) and preinfundibular (Patients 6 and 8) variants of craniopharyngioma growth seem to be associated with the highest possibility of preservation of the stalk function. Longitudinal incision along portal vessels of the stalk after extracapsular dissection and surface observation may be the safest introduction into stalk-sparing surgery. However, in transinfundibular craniopharyngiomas (originating from the inside of the stalk and producing stalk expansion), the GTR can be achieved only with stalk resection.^2^ This new panhypopituitarysm is not necessarily accompanied with diabetes insipidus in all cases.^37^

The overall rate of new endocrinopathy in craniopharyngioma surgery is reported at 37% for transcranial approaches and 25.7% in small series using EEA (Table 4).^18^–^24^ Our results are comparable to the other few series of suprasellar craniopharyngiomas, removed by EEA, except for the incidence of new endocrinopathy and CSF leak (Table 4). New endocrinopathy (including DI) appeared in 5/8 (62.5%) patients. Stalk was preserved in 4 patients, resulting in normal endocrine status in 2 and partial hypopituitarism in one and panhypopituitarism in one of them. The preservation of the continuity of the stalk in extraventricular cranio pharyngiomas enables the pituitary function, however, traction injury to the stalk and its intrinsic vessels or hypophyseal arteries can result in complete or partial pituitary failure. Vasospasm may also be responsible for some failures in pituitary function despite stalk preservation. In Patient 6 (Figure 4b; Supplement 1), the pituitary-suprarenal axis has recovered after 6 months, but not in other patients with partial stalk preservation (Patients 3 and 7). Only one patient had preexistent panhypopituitarism (Patient 4), but got new DI. Our results are at least partially influenced by a higher number of transinfundibular tumours, where stalk preservation is less likely.

### Hypothalamic injury and quality of life

Cognitive, behavioral and social problems and re-employment rates are often neglected among surgical results in either transcranial or trans-sphenoidal series of adult patients with craniopharingioma, but are well-known in pediatric population.^4^,^10^,^24^ The quality of life after craniopharyngioma surgery is determined by cognitive problems from hypothalamic injury and visual impairment. Infiltration of the hypothalamus and ill-defined cleavage plane are the main intraoperative circumstance that may preclude partial resection to avoid hypothalamic injury. Hypothalamic injury is also possible in extraventricular craniopharyngiomas from the firm adeherence of the tumour to the hypothalamic surface and perforators (Figure 4, Supplement 1).

Hypothalamic injury can be lethal. Memory problems and psycho-organic change are the most disabling symptoms that affect re-employment and return to school. Up to 84% of patients can live independent life with social integration and retain professional occupation after transcranial surgery.^31^ Obesity, hyperphagia and sleep disorders also represent hypothalamic injury. These were initially detected in 4/8 our patients (Patients 3, 4, 5 and 6). Petechiae of the inner walls of the infundibulum can be seen in Figure 2d, resulting from forced blunt dissection of the capsular dome (Supplement 2). Similarly, the subpial bleeding of the left outer hypothalamic surface resulted from forced blunt capsular detachment (Patient 6, Figure 1 (4b); Supplement 2). In this patient, unilateral millimeter-sized ischemic lesion in the deep of the left hypothalamus can be seen on the postoperative MRI (Patient 6, Figure 1 (4c)), possibly produced by traction of the hypothalamic perforators. Both patients (Patients 5 and 6) developed psycho-organic change and circadian rhythm sleep disorder, which became apparent in the second postoperative week; these patients presented with severe daytime somnolence. This symptomatic narcolepsy in some patients after removal of craniopharyngioma was found to be related to the low CSF hypocretin-1 level, resulting from injury to the hypocretin-producing neurons in the hypothalamus.^40^ Sleep disorder was temporary in both patients and disappeared spontaneously in few months.

Two of 4 patients with postoperative hypothalamic dysfunction never regained their previous quality of life because of psycho-organic change and memory problems (Patients 3 and 6, but disappeared in Patients 4 and 5; Table 2). Additionally, one patient with no cognitive problems did not retain his occupation because of new visual field impairment in one eye (Patient 7; disturbing inferior half visual fields loss).

### Cerebrospinal leak

CSF leak is the single most discrepant complication when comparing transcranial and endonasal approaches to craniopharyngiomas. This major drawback of transplanum transtubercle approach has been recently significantly reduced. The average rates from a small number of series, published in the last 5 years is about 24.7% (range 3.8–68%).^18^–^24^ However, Jane *et al*. in 2010 and Leng *et al*. in 2012 reported rates of 0% and 3.8%, respectively.^23^,^24^ Leng *et al*. suggested to use the gasket seal closure using fascia lata with Hadad’s flap over it and sealant. Although effective closure techniques were published in 2006, these were not used in the first half of our cases. The CSF leak rate in our first 8 patients was 2/8 (25%) patients (Table 4), while there was no CSF leak in the last 3 patients, using Hadad’s flap. However, the routine use of CSF diversion postoperatively for approximately 10 days has not yet been abadonded by us. There were no subdural effusions or other complications using lumbar drainage of 250–300 mL of CSF outflow per day. Despite exposure of the third ventricle in some of our patients, postoperative antibiotics were not used routinely, except as a single bolus of cephalosphorine (2g) 30 minutes before surgery and after 3 hours of surgery if applicable. Two patients developed meningitis on postoperative day 14 (Patient 4; no bacteria) and 22 (Patient 5; *Pseudomonas aeruginosa*), respectively. Both were treated succesfully with antibiotics, but implantation of a ventriculo-peritoneal shunt was necessary to treat the hydrocephalus. We suggest immediate revision in recurrent CSF and a shorter lumbar drainage time to decrease the rate of CSF infection and hydrocephalus.

### Residual tumours and radiation therapy

The management of a remnant tumour is also a point of controversy. A significant number of patients with residual tumours remain stable for a long time.^4^ There is a growing evidence in recently published data that similar tumour control rates with less hypothalamic and hypophyseal morbidity can be achieved with STR combined with fractioned radiotherapy (fXTR) or radiosurgery (SRS) as compared to GTR.^10^,^11^ Yang *et al*. studied control rates of different treatment modalities in 442 patients.^11^ Progression free survival at 2 and 5 years did not differ between GTR (88 *vs.* 91%) and STR + XRT (67 *vs.* 69%) subgroups. Similar overall survival rates were found at 5 and 10 years (98 *vs.* 99% and 98 *vs.* 95%).^11^ In meta-analysis by Sughrue *et al*., 540 patients, treated for craniopharyngioma (mean follow-up >4 years) were stratified into GTR, STR, STR + XTR, fXRT and SRS subgroups.^10^ Overall, new endocrinopathy was expected in 37% and overall visual decline in 3.7%. All irradiated patients showed 2-fold increased rate of visual decline as compared to GTR alone. In contrast, GTR was associated with new endocrinopathy 2.5-fold more often (52%) than STR or STR+ XRT. Interestingly, adjuvant XTR in STR alone was not significantly related to visual deterioration.

The EEA may significantly improve the results either in GTR or STR (+ XRT) groups.

### Transcranial approaches

Transcranial approaches include 9–90% GTR rate (on average 60–80%).^9^,^14^–^16^,^31^,^32^,^41^ The transcranial approaches represent principally lateral, anterior and superior routes to craniopharyngiomas. Transpetrosal approach enables posterior route to retrochiasmatic region. A combination of approaches is applied in 10% of cases.^15^,^17^,^33^ In the most commonly used pterional-transsylvian approach, certain amount of manipulation and mechanical stress to the contralateral neurovascular structures and blind resection are included.^9^,^14^–^16^ The neurosurgeon removes the tumour between both optic nerve, optic nerve and the supraclinoid ICA, lateral to ICA and superior to ICA bifurcation. Intraventricular part of the tumour is approached anteriorly by trans-lamina terminalis (suitable in cases with a prefixed chiasm) or superiorly by interhemispheric transcallosal approaches. Basal trans-lamina terminalis approach is a midline transcranial approach which is the closest approximation to EEA.

As some recent transcranial series exist with similar high GTR rate over 80%, however, the comparisson between transcranial and endonasal cases in Table 4 is difficult, because transcranial series include complex and multicompartment craniopharingiomas.^1^,^15^,^17^,^34^ The sum of >95% for GTR and NTR together has been reported with EEA recently (Table 4).^24^ The advantage of direct bilateral visualization with EEA might become more apparent in functional outcomes and decreased major neurological risks (hemiparesis, cranial nerve deficit, intracranial hematoma, etc.) in more calibrated comparative studies in the future.

### Utility and limitations

EEA is indicated to strictly midline supradiaphragmatic craniopharyngiomas. If GTR is the primary goal, extension lateral to ICA bifurcation and lateral to the optic nerve and tract are contraindications to EEA.^2^ The advantages of EEA are mostly apparent in small to mid-sized centrally protruding supradiaphragmatic craniopharyngiomas. The intraventricular part of the tumour is also easily removable after passing the cleavage line at the infundibulum. Direct visualization in combination with bimanual microsurgical technique enables circumferential and more controlled tumour removal at the cleavage line bilaterally and perforators preservation, stalk-sparing surgery, better and earlier judgement of hypothalamic involvement and earlier switch to subtotal resection. The chances to preserve the integrity of the hypothalamus-stalk-pituitary complex and vascularity of the hypothalamo-chiasmatic region increase with direct multiangled inspection provided by the endoscopy-aided microsurgery.

However, large and giant tumours with lateral extensions into the basal ganglia and middle fossa, retroclivally, into cerebellopontine angle and towards foramen magnum or largely anteriorly projecting tumours or encircling neurovascular structures should be accessed with transcranial approaches.^2^, ^9^, ^14^, ^22^

All authors agree that pure intraventricular craniopharyngiomas (a rare entity) are not an indication for EEA, which would traverse and damage the functioning hypothalamus.^2^,^3^,^6^,^9^,^21^,^27^ Because morbidity and mortality dramatically increase in reoperations for recurrent craniopharyngiomas, EEA is the first method of choice in recurrent and residual craniopharyngiomas after transcranial surgery, providing inferior “virgin” route as stated by Cavallo *et al*.^19^

## Conclusions

Our early outcome results are comparable to the recent few series of patients with supradiaphragmatic craniopharyngiomas, removed by EEA, except for the incidence of new endocrinopathy and CSF leak rate (Table 4). This was influenced by a higher number of transinfundibular tumours in our series, where stalk preservation is less likely, and not using nasoseptal flap or gasket closure in the first half of cases.

There is a tendency of improved GTR rate with less morbidity in endonasal approaches as compared to transcranial approaches.^11^,^20^,^21^,^24^ This is more obvious for visual outcome and quality of life than for endocrine outcome.^2^,^10^,^16^–^18^,^20^–^24^ There is no current data of outcomes yet stratified for certain morphological subtypes of craniopharyngioma. It is intuitively expected in the future, that infradiaphragmatic and suprasellar extraventricular craniopharyngiomas will be agressively approached for GTR with EEA, while infundibulum-invading craniopharyngiomas will be treated with subtotal endoscopical resection and XTR. The first hundred of patients, published to date in a few series and including ours, have been operated using endoscopic transplanum-transtuberculum approach for newly diagnosed or recurrent and residual supradiaphragmatic craniopharyngiomas. The cumulative results of the last 8 series are promising, however, these hypotheses have to be tested on larger number of patients in the future, considering also a postoperative quality of life.^2^,^19^–^24^,^41^

## Figures and Tables

**TABLE 1.** Clinical features of 8 patients with supradiaphragmatic craniopharingioma operated on by transplanum approach

**Pt. No.**	**Sex, age**	**Signs and symptoms**			**Duration (months)**	**Tumor size (mm) W × H × L**	**Tumor Grade**					**Follow-up period**
	
		**Leading symptom(s)**	**Visual Right/Left eye**	**Endocrine function**			**Hoffman [ref. 13]**	**Samii [ref. 16]**	**Yasargil [ref. 17]**	**Kassam [ref. 26]**	**Songtao Qi [ref. 5]**	
1	M, 66	Visual	BTH, ↓acuity	Normal	6	15×15×20	Subch	II	Suprasel	Retroinf	Extrav	5yr 9mo
2	M, 73	Visual, retrobulb. HA	Sup. BTQ	Normal	2,5	25×25×15	Intrav	III	Intrav	Transinf	Transinf	4yr
3	M, 51	HA, balance, psih	Normal	Normal	4	27×31×25	Intrav	IV	Intrav	Transinf	Extrav & Intrav	3yr 4mo
4	F, 71	V&N, visual	TH/Sup. TQ, ↓acuity L	Panh, no DI	4	17×34×21	Intrav	IV	Intrav	Transinf	Transinf	2yr 9mo
5	F, 60	Visual	BTH R > L	Normal	6	21×16×16	Retroch	III	Extrav	Preinf	Extrav & Intrav	1yr 9mo
6	F, 69	Visual	Sup. TQ/central scotoma, ↓acuity L	Normal	5	25×21×17	Retroch	III	Extrav	Preinf	Extrav & Intrav	18mo
7	M, 57	Visual	Inf. BTQ scotomas	Normal	5	22×20×15	Retroch	III	Extrav	Preinf	Extrav & Intrav	13mo
8	F, 47	Visual	Inf. NQ scotoma L	Normal	2	20×29×20	Subch	II	Suprasel	Preinf	Extrav	10mo

HA = headache; V&N = vomitus and nausea; BTH = bitemporal hemianopsia; BTQ = bitemporal quadrantanopsia; NQ = nasal quadrantanopsia; Panh = panhypopituitarysm; WxHxL = width, height, lenght; DI = diabetes insipidus; Subch = subchiasmatic; Retroch = retrochiasmatic; Intrav = intraventricular; Extrav = extraventricular; Suprasel = suprasellar; Preinf = preinfundibular; Retroinf = retroinfundibular; Transinf = transinfundibular

**TABLE 2.** Visual, endocrine, neurological and cognitive outcomes in eight patients with craniopharingioma operated on by transplanum approach

**Pt. No.**	**Sex, age**	**Visual outcome**	**Visual postop status**	**Endocrine outcome**	**Permanent diabetes insipidus**	**Stalk preservation**	**Neuro/cognitive consequences**	**Occupational/life style resumed**	**Tumor removal**

		**Right/Left**	**Right/Left**						
1	M,66	Normalised	Normal	Normal	No	Yes	No	Yes	GTR
2	M, 73	Normalised	Normal	Panh	Yes	No	No	Retired (same)	GTR
3	M, 51	Normal (same)	Normal	Panh	Yes	No	Obesity +30kg, memory, psih	No	GTR
4	F, 71	Improved	can read L+R, less bilat TH	Same (Panh)	Yes (triple response)	No	Mild psih (transit) Obesity +20kg	Retired (same)	NTR
5	F, 60	Improved R/Worse L	R less temporal/L TH + sup.NQ, can read L+R	Panh	Yes	No	Sleep (transit), mild psih (transit)	Housewife (same)	GTR
6	F, 69	Improved	can read L+R, R normal / L less central scotoma	Partial hypopituit.	No	Yes	Sleep (transit), psih, memory, obesity +15kg	No	GTR
7	M, 57	Improved R/ Worse L	R less inf.TQ/L temporal complete	Panh	Yes	Yes	No	No	STR
8	F, 57	Normalised	Normal	Normal	No	Yes	No	Yes	GTR

Newly aquired visual deficit are undelined; TH = bitemporal hemianopsia; NQ = nasal quadrantanopsia; TQ = temporal quadrantanopsia; Panh = panhypopituitarism; hipopit = hypopituitarism; psih = psihoorganic symptomatology; transit = transitory; GRT = gross total removal; NTR = near total removal; STR = subtotal removal

**TABLE 3.** Sellar closure techniques and complications in transplanum transtuberculum approach for craniopharyngioma

**Pt. No.**	**Sellar closure technique**	**Lumbar drainage (days)**	**CSF leak**	**Revision**	**Revision technique**	**Meningitis**	**Hydrocephalus / internal drainage**
1	Neuropatch bilayer, Beriplast, Spongostan	10	No	None		No	No
2	Neuropatch bilayer, Beriplast, Sph obliteration (fat)	14	No	None		No	No
3	Neuropatch inlay, Beriplast, Neuropatch overlay, Tachosil, Spongostan	12	No	None		No	No
4	Neuropatch inlay, Tachosil overlay, Spongostan, Beriplast	11 (+13)	Yes	Twice	I. topic obliteration (Tachosil), Beriplast II. topic obliteration (fat), FL overlay, Sph obliteration (fat, Beriplast)	Yes (no bacteria)	Yes
5	Neuropatch bilayer, Tachosil, Hadad, Duraseal	20 (+14)	Yes	Once	Topic obliteration (fat), Beriplast, Sph obliteration (fat).	Yes (Ps. aer.)	Yes
6	Fat intrasell, FL bilayer, Hadad, Duraseal	12	No	None		No	No
7	Fat intrasell, FL bilayer, Hadad, Duraseal	11	No	None		No	No
8	Tachosil inlay, Duraform overlay (double), Tachosil overlay, Hadad, Duraseal	16	No	None		No	No

FL = fascia lata; Sph = sphenoid sinus; Neuropatch = microporic polyesther urethane (B. Braun Melsungen AG, Melsungen, Germany); Beriplast = fibrin glue CSL Behring, King of Prussia, Pennsylvania, USA); Duraseal = synthetic sealant (Covidien, Dublin, Ireland); Tachosil = animal derived collagen sponge with fibrinized surface (Takeda Pharmaceuticals International GmbH, Zurich, Switzerland); Spongostan = cellulose sponge (Ethicon Biosurgery, Somerville, New Jersey, USA); Hadad = nasal septal vascularized flap; Duraform = collagen-based biocompatible dural implant (Codman, Raynham, Maryland, USA); Ps. aer. = Pseudomonas Aeruginosa

**TABLE 4.** Literature review of the outcomes in extended endonasal aproach for craniopharyngioma

**Author, year, [reference]**	**No. of cases**	**GTR / NTR (%)**	**Vision improvement (%)**	**New DI / Panhypopituitarism (%)**	**Cognitve decline (%)**	**Occupational /life style Resumed (%)**	**CSF leak rate (%)**
Frank et al., 2006, [21]	10	70/0	75	30/0	na	na	30
De Divitis et al., 2007, [20]	10	70/0	71	43/17	na	na	20
Gardner et al., 2008, [22]	16	50/25	93	8/18	na	na	69
Cavallo et al., 2009, [19]	22 (all reop.)	41/36	83	14/40	na	na	14
Campbell et al., 2010, [18]	14 (all new)	20/36	79	8/0	na	na	36
Jane et al., 2010, [23]	12	42/42	78	44/67	na	na	0
Leng at al., 2012, [24]	26	69/8 (86/19)*	77	42/38	12	69	3.8
Ikeda et al., 2012, [31]	15	60/0	93	20/67	na	na	0
Bošnjak *et al, current series*	8 (all new)	75/13*	75	63/57	25	63	25

GTR = gross total removal; NTR = near total removal; DI = diabetes insipidus; CSF = cerebrospinal fluid; reop = reoperation for residual or reccurent tumor; * primarily GTR attempted, na = data not available.

## Supplements

Supplement 1: Patient No. 6 http://www.degruyter.com/view/j/raon.ahead-of-print/raon-2013-0036/10019_EB_2013_06_04_06_video_1_Patient_6.mov

Supplement 2: Patient No. 5 http://www.degruyter.com/view/j/raon.ahead-of-print/raon-2013-0036/10019-EB-2013-06-04_06_video_2_Patient_5.mov

Supplement 3: Patient No. 8 http://www.degruyter.com/view/j/raon.ahead-of-print/raon-2013-0036/10019-EB-2013-06-04_06_video_3_Patient_8.mov
